# Evaluation of nerve function after Bell’s palsy based on different facial nerve assessment scales HBGS/SFGS/MPS: A comparative study

**DOI:** 10.1371/journal.pone.0326789

**Published:** 2025-06-25

**Authors:** Zhanxiang Lin, Xiaoyang Fei, Lichao Huang, Yuchun Shao, Zicai Liu

**Affiliations:** 1 Department of Rehabilitation Medicine, Shaoguan First People’s Hospital, Shaoguan, Guangdong Province, China; 2 Department of Rehabilitation Medicine, Yuebei People’s Hospital, Shaoguan, Guangdong Province, China; Universiti Malaysia Pahang Al-Sultan Abdullah, MALAYSIA

## Abstract

**Background:**

Studies have shown that approximately 30% of patients with Bell’s palsy may experience permanent disfigurement due to muscle weakness affecting facial expressions. The prognosis for Bell’s palsy is often correlated with the degree of impairment in facial nerve function observed in the early stages of the condition. Selecting appropriate assessment tools is essential for the effective treatment and prognosis of patients with Bell’s palsy.

**Objective:**

Examine and compare three scales used to measure Bell’s palsy: the House-Brackmann Grading Scale (HBGS), the Sunnybrook Facial Grading System (SFGS), and the Modified Portmann Scale (MPS).

**Methods:**

A retrospective study involved 97 patients with Bell’s palsy treated at the authors’ institution from 2021 to 2022. The authors accessed these data for research purposes in February 2025. The patient cohort underwent HBGS, SFGS, and MPS to assess facial nerve function before and after treatment. We compared the results of these three evaluation methods. Additionally, we performed correlation analyses and receiver operating characteristic (ROC) analyses of the post-treatment data to examine the relationships between HBGS and SFGS and between HBGS and MPS, using the internationally recognized and most widely utilized HBGS as the standard.

**Results:**

The HBGS demonstrated moderate consistency with both the FSGS and MPS, revealing significant negative correlations (r = −0.876, P < 0.01; r = −0.860, P < 0.01). All three scales exhibited high reactivity and showed no ceiling/floor effect. The optimal cut-off values for the FSGS and MPS were determined to be ≤ 68 and ≤ 16 points, respectively, with AUC of 0.948 (95% CI: 0.910–0.984) and 0.931 (95% CI: 0.883–0.968).

**Conclusion:**

HBGS, SFGS, and MPS are all appropriate tools for assessing Bell’s palsy. The severity of facial nerve palsy can be clinically classified using a transect score of 68 on the SFGS and 16 on the MPS. However, the results should be interpreted with caution, as objective indicators were not utilized as criteria.

## 1. Introduction

Bell’s palsy, also known as idiopathic facial nerve palsy, is a prevalent cranial nerve disorder. It is characterized by acute paralysis of the ipsilateral seventh cranial nerve, resulting in paralysis or weakness of the muscles on one side of the face [1]. The annual incidence of Bell’s palsy has been reported to be approximately 0.02% to 0.03%, with a recurrence rate of up to 12% [2,3]. There is also evidence of a genetic predisposition to Bell’s palsy [4]. Additionally, patients with Bell’s palsy commonly experience clinical symptoms such as mild fever, pain behind the ears, taste disturbances, auditory hypersensitivity, facial changes, drooling, and dry eyes [3]. Additionally, patients with Bell’s palsy commonly experience clinical symptoms such as mild fever, pain behind the ears, taste disturbances, auditory hypersensitivity, facial changes, drooling, and dry eyes [5]. Approximately 30% of patients may experience permanent weakness in the facial expression muscles, resulting in disfigurement [6]. Despite its seriousness, the exact etiology of Bell’s palsy remains unclear [7]. A variety of factors may contribute to its development, including the anatomy of the facial nerve, viral infections, ischemia, immune inflammation, and acute cold exposure [7–9]. These potential etiologies undoubtedly have a profound impact on treatment strategies. Inadequate or delayed treatment can significantly hinder the patient’s recovery process and may lead to long-term sequelae or even permanent damage [7,10,11]. Therefore, it is particularly important to implement effective interventions for facial rehabilitation, and the foundation for successful management is an accurate and efficient assessment of the functional status of the facial nerve.

How to scientifically select the appropriate scale based on the study’s purpose is the initial challenge faced in evaluating facial nerve function in clinical research. There are more tools used internationally to evaluate facial nerve palsy, but there have been no definitive studies to show which scale is most appropriate, which has led to inconsistencies in their use, as well as differences in validation and clinical applicability [12–15]. The House-Brackmann Grading Scale (HBGS) is an internationally recognized and widely used method for assessing facial nerve palsy, known for its good reliability and ability to visually evaluate the condition [16]. However, it may not accurately reflect subtle changes, such as joint movements and facial contractures [17]. The Sunnybrook Facial Grading System (SFGS) appears to be gaining popularity among researchers and has been recommended for assessing facial nerve palsy [18]. Additionally, the Modified Portmann Scale (MPS) is a commonly used tool for evaluating facial nerve function, particularly among Chinese scholars [19–23]. Compared to the HBGS, the MPS may offer a more objective assessment of joint band movements.

The three scales have some differences in dimensionality and level setting (e.g., evaluation of total scores and items), and thus measurements may differ when used in the same population. For example, the HBGS may be more cursory [24], whereas the SFGS may be relatively complex. Currently, there are no tests or comparative studies on the performance of MPS. In addition, the facial bones, muscle distribution, cultural background and expression habits of the Chinese population are somewhat different from those of the Western population, which may also impact the results. Therefore, in this study, the three scales were used in Chinese patients with Bell’s palsy, and the internationally recognised HBGS was used as the standard to compare and assess the aspects of correlation, sensitivity and specificity with SFGS and MPS. Also, the reactivity of the three scales was compared, and the effects of ceiling/floor were calculated. This study aims to provide new performance evidence for clinical practice and the measurement of core outcome indicators in Bell’s palsy by evaluating the facial paralysis assessment scale using statistical methods such as measuring correlation, calculating reactivity and the effects of ceiling/floor, and estimating sensitivity and specificity.

## 2. Methods

### 2.1. Study design

This is a retrospective, comparative study. The study protocol received approval from the Ethics Committee of Yuebei People’s Hospital (approval number: KY-2021–075).

### 2.2. Participants

This study reviewed the medical records of patients with Bell’s palsy who attended the inpatient and outpatient departments of rehabilitation medicine and neurology at a tertiary care hospital in the Shaoguan region between 2021 and 2022. The initial diagnosis of the disease is based on electromyography and electro-neurography, supplemented by imaging and laboratory tests if necessary, and finally determined by the Chief Physician of the Hospital’s Department of Rehabilitation Medicine, who has more than 30 years of clinical experience. These data were accessed in February 2025. The researcher screened for eligibility for inclusion, as detailed below:

Inclusion Criteria: 1) A diagnosis of Bell’s palsy made by an experienced clinician; 2) Age range of 18–75 years; 3) First occurrence of unilateral facial paralysis; 4) Onset within one month, with all initial HBGS assessments rated at grade III or above [25]; 5) Ability to maintain consciousness, demonstrate reading comprehension, and express verbally; 6) Patients who are willing to participate and have completed two rounds of HBGS, SFGS, and MPS assessments.

Exclusion Criteria: 1) Facial nerve palsy resulting from other causes, including Lyme disease, encephalitis, tumors, or trauma; 2) Psychiatric disorders, such as major depression or cognitive impairments; 3) Inability to complete a course of clinical treatment.

### 2.3. Procedures

The investigators conducted two rounds of assessments for all patients, utilizing HBGS, SFGS, and MPS to evaluate facial nerve function at the onset of the disease and again after two weeks of clinical treatment. The time interval between the three assessment tools did not exceed one minute for each patient. The assessment of all scales was conducted independently by two professionals at our hospital, and the final results were determined as the average of the scores from these two assessors. The assessment data from both rounds were used to calculate and compare the reactivity and ceiling/floor effects of the three scales. Data from the second round of assessments were employed to compare the correlations between the HBGS and SFGS, as well as between the HBGS and MPS. Additionally, the predictive performance of the SFGS and MPS was analyzed and evaluated using ROC curves, with the HBGS serving as the standard.

### 2.4. Measures

#### 2.4.1. House-brackmann grading scale (HBGS).

HBGS facial paralysis is classified into six grades: Grade I indicates normal function, with full movement in all facial areas, scoring 1 point. Grade II represents mild functional impairment, characterized by slight facial muscle weakness, potentially accompanied by diminished joint band movements and mild asymmetry at the corners of the mouth, scoring 2 points. Grade III denotes moderate functional impairment, with noticeable facial muscle weakness but no facial deformity, and mild asymmetry at the corners of the mouth upon maximal effort, scoring 3 points. Grade IV indicates moderate to severe functional impairment, with significant facial muscle weakness or evident facial deformity, resulting in pronounced asymmetry at the corners of the mouth, scoring 4 points. Grade V reflects severe functional impairment, with nearly imperceptible facial movements and only slight movement at the corners of the mouth, scoring 5 points. Grade VI signifies complete paralysis, with no facial movement whatsoever, scoring 6 points [26]. A higher score indicates a more severe facial nerve injury.

#### 2.4.2. Sunnybrook facial grading system (SFGS).

The evaluation of SFGS encompasses static symmetry, the symmetry of autonomous motion, and associative motion [27]. The scale is derived by comparing the paralyzed side of the patient with the healthy side, resulting in a total score that ranges from 0 to 100. Higher scores indicate better facial nerve function [28]. Additionally, Supporting information 1 contains figures of the English and Chinese versions of the scale (S1 and S2 Fig).

#### 2.4.3. Modified portmann scale (MPS).

The MPS consisted of six items: smiling, frowning, closing the eyes, whistling, moving the nose wing, and puffing out the cheeks. Each item was worth 3 points, while the impression of quietness was assigned 2 points (This score is used to assess the symmetry of the subject’s lower face at rest and is categorised as: excellent (2 points, normal symmetry), good (1 point, mild asymmetry) and poor (0 points, marked asymmetry)), resulting in a total possible score of 20 points. The scoring criteria were as follows: a score of 3 points indicated movement similar to the healthy side, 2 points indicated reduced movement, 1 point indicated slight voluntary movement, and 0 points indicated no voluntary movement. The quiet impression score was categorized as follows: excellent (2 points), good (1 point), and poor (0 points) [19]. Higher scores reflect better facial function.

### 2.5. Data analysis

All Statistical analyses were performed using R version 4.2.3 and Python version 3.11.4. Count data were analyzed descriptively. Firstly, through the Kolmogorov-Smirnov test, we determined that the data conformed to an approximately normal distribution. The correlations between the scales were analyzed using the Bland-Altman and Pearson methods. The Bland-Altman method calculates the 95% consistency limits of the results from the two methods and graphically represents these limits to determine whether there is consistency between them. The r-value ranges from −1–1, with larger absolute values indicating stronger correlations. A correlation of 0.1 to 0.3 is considered weak, 0.3 to 0.5 is moderate, and 0.5 to 1.0 is strong. Reactivity was utilized to compare the scale scores before and after the intervention using the paired sample t-test, with a P-value of less than 0.05 deemed statistically significant. The response was evaluated by integrating effect size (ES) and standardized response mean (SRM). Absolute values of ES and SRM around 0.2 indicate low responsiveness, around 0.5 indicate medium responsiveness, and 0.8 or above indicate high reactivity [29]. In addition, the proportion of the highest and lowest total scores was calculated to evaluate the ceiling or floor effect. If the ratio exceeds 15%, the total score is considered to exhibit a ceiling or floor effect [30]. Finally, the predictive performance of SFGS and MPS was analyzed by plotting ROC curves, using HBGS as the standard.

## 3. Results

### 3.1. General information

A total of 97 patients with Bell’s palsy were included in the analysis, consisting of 48 males and 49 females, with ages ranging from 19 to 73 years. Among these patients, 45 exhibited left-sided facial paralysis, while 52 had right-sided facial paralysis. The duration of the condition varied, with the shortest recorded at 1 day and the longest at 27 days.

### 3.2. Correlation analysis of facial nerve function assessment scales

**Fig 1:**
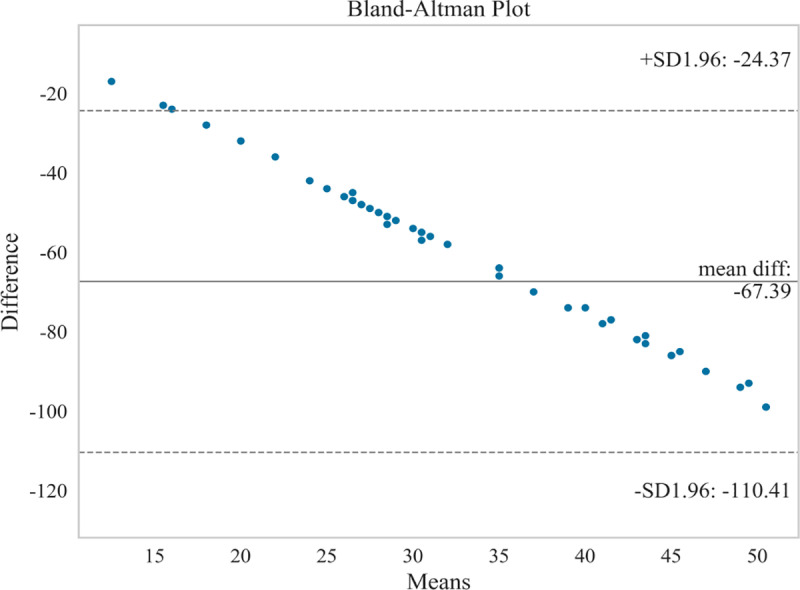
Bland-AItman plot between HBGS and SFGS.

**Fig 2:**
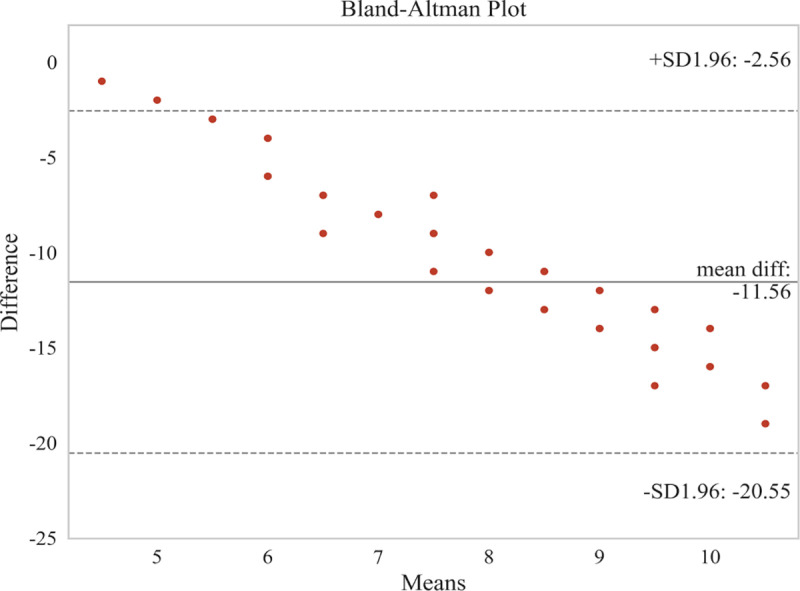
Bland-AItman plot between HBGS and MPS.

In this study, the Bland-Altman and Pearson methods were employed to analyze the correlation between HBGS and SFGS, as well as between HBGS and MPS. The Bland-Altman analysis results indicated the following: 1) The upper and lower limits of consistency for HBGS and SFGS were −24.37 + 1.96 and −110.41–1.96, respectively (Fig 1), with a correlation coefficient of r = −0.876 (P < 0.01); 2) The upper and lower limits of consistency for HBGS and MPS were −2.56 + 1.96 and −20.55–1.96, respectively (Fig 2), with a correlation coefficient of r = −0.860 (P < 0.01). Bland-Altman analysis calculates 95% limits of agreement (mean difference ± 1.96 × SD), where narrower limits indicate stronger absolute agreement [31,32]. In our study, the limits of agreement widths (e.g., −24.37 to −110.41 for HBGS vs. SFGS) were interpreted as moderate consistency, reflecting variability exceeding ideal clinical precision thresholds [15,16]. Consequently, the above results demonstrate that HBGS exhibited moderate agreement with both SFGS and MPS, along with a significant negative correlation.

### 3.3. Reactivity analysis of facial nerve function assessment scales

The reactivity results (Table 1) indicated that the scores of the three scales before and after treatment exhibited statistically significant differences (P < 0.01). The ES and SRM demonstrated that all three scales exhibited high reactivity.

**Table 1:** Scale reactivity analysis.

scale	pre- intervention^*^	post- intervention^*^	difference value^*^	P-value	ES	SRM
HBGS	4.64 ± 0.60	2.54 ± 0.83	2.10 ± 0.82	<0.01^**^	3.50	2.56
SFGS	27.00 ± 14.60	69.93 ± 21.33	−42.93 ± 18.18	<0.01^**^	−2.94	−2.36
MPS	5.49 ± 2.34	14.09 ± 3.88	−8.60 ± 3.47	<0.01^**^	−3.68	−2.48

* Mean ± SD;

** Paired t test.

### 3.4. Ceiling and floor effects of facial nerve function assessment scales

The HBGS raw scores ranged from 1 to 6, with the highest score being 1 and the lowest score being 6; the SFGS raw scores ranged from 0 to 100, with the highest score being 100 and the lowest score being 0; and the MPS raw scores ranged from 0 to 20, with the highest score being 20 and the lowest score being 0. The lowest scores on all three scales were 0% of patients, with 5.7% of patients having the highest HBGS and SFGS scores and 5.2% of patients having the highest MPS scores. The results showed that there was almost no floor or ceiling effect for the three scales.

### 3.5. ROC curve analysis of facial nerve function assessment scales

**Fig 3:**
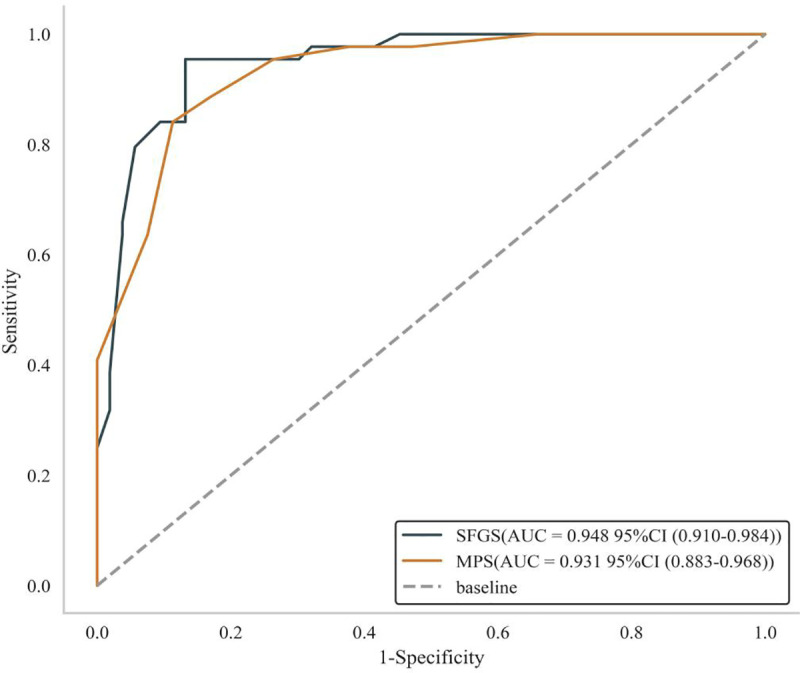
ROC curves of SFGS and MPS.

Studies have shown [33] that an HBGS score of less than 3 indicates a good prognosis or complete recovery, while a score of 3 or higher suggests incomplete recovery. We utilized the internationally recognized HBGS as a predictive criterion, establishing a cut-off score of 3. The data were transformed into dichotomous variables, and ROC curves were plotted to evaluate the efficacy of SFGS and MPS in diagnosing the degree of facial nerve palsy (severe/moderate = 1, mild = 0). Table 2 below demonstrates that the optimal cut-off values for SFGS and MPS were ≤ 68 and ≤ 16 points, respectively, using an HBGS score of 3 or higher as the criterion. The AUCs were 0.948 (95% CI: 0.910–0.984) and 0.931 (95% CI: 0.883–0.968), respectively (Fig 3).

**Table 2:** Diagnostic efficacy analysis of the scales.

Name	Sample	AUC	Optimal cut-off value (score)	TPR(%)	TNR(%)	Youden’s Index
SFGS	97	0.948	68	95.5	86.8	0.822
MPS	97	0.931	16	84.1	88.7	0.728
TPR, True Positive Rate; TNR, False Positive Rate.						

## 4. Discussion

### 4.1. The findings of this study

In this study, we compared the reactivity of the HBGS, SFGS, and MPS in assessing the condition of Chinese patients with Bell’s palsy both before and after treatment, as well as their ceiling and floor effects. Additionally, we used the HBGS as a reference standard to evaluate its correlation with the SFGS and MPS, and to predict the diagnostic performance of the latter two, including sensitivity and specificity. To our knowledge, no study of this nature has been conducted previously. The results of this study demonstrated moderate consistency and a significant negative correlation between the HBGS and SFGS, as well as between the HBGS and MPS (correlation coefficient r was close to −1, P < 0.01). This finding is expected, as higher HBGS scores indicate more severe symptoms, while SFGS and MPS scores reflect the opposite, suggesting a strong correlation between them. In terms of reactivity, the three scales exhibited heightened responsiveness. There was no ceiling or floor effect, indicating that these scales possess a good degree of differentiation. Through AUC analysis, this study revealed that SFGS and MPS exhibited excellent predictive performance (AUC > 0.9), with high sensitivity and specificity. This further suggests that SFGS and MPS may be comparable to HBGS in assessing symptoms of Bell’s palsy. Notably, SFGS demonstrated slightly superior predictive performance, with an AUC closer to 1. Overall, we conclude that these three scales are suitable for patients with Bell’s palsy, and that SFGS and MPS are equally effective as HBGS in evaluation.

### 4.2. Progress and comparison of previous studies

In each case of facial palsy, efficient grading of disease severity is essential for early implementation of appropriate treatment and follow-up to assess the course of the disease [34]. Lewis et al. [13] concluded that an excellent facial palsy grading system should have three characteristics: 1) the ability to assess the severity of facial palsy and ensure highly reproducible and consistent results; 2) the ability to track the patient’s recovery process; and 3) simplicity. The HBGS was adopted by the American Academy of Otolaryngology, Head and Neck Surgery, as the standard for the assessment of facial nerve function in 1985 [24]. However, it has been pointed out that the HBGS is a crude scale that may not be effective in determining changes in facial nerve function after therapeutic intervention [15]. Indeed, when we look at the grading of the scale, for example, grade III of the HBGS is defined as “normal symmetry, but with significant muscle weakness and possibly associated joint band movements or hemifacial spasms,” while grade IV is directly described as “significant weakness or asymmetry,” but the actual functional difference between the two is difficult to quantify. The actual functional difference between the two is difficult to quantify. Nevertheless, due to the scale’s ease of use and wide clinical acceptance, it is difficult to replace the scale in the short term [17].

Kanerva et al. [18] compared the clinical applicability of the HBGS, Facial Nerve Grading System 2.0 (FNGS), and SFGS in the assessment of facial nerve palsy and demonstrated that the SFGS was the most applicable as it was able to regionally assess variables such as voluntary movement, static symmetry, and joint banding phenomena. Previous studies have shown [35] that the SFGS demonstrates a high degree of reproducibility among experienced clinicians and novices, with minimal inter- and intra-rater variability, as well as excellent internal validity and sensitive change detection. The results of the present study are in agreement with them, with a high correlation between the SFGS and the HBGS and a sensitive response to neurological recovery on the face. Although there were 2 independent evaluators, each performed all three assessments, which might also explain the high correlation between them. However, it has also been noted [36] that SFGS scores are highly variable and not very stable in assessing linkage movements due to the complexity and difficulty in observing facial linkage movements. In addition, the relatively more complex assessment process has also hindered the widespread use of SFGS [37].

Currently, there are relatively few international studies utilizing the MPS to evaluate facial nerve function. This type of scale focuses on facial movements [38]. Unlike the HBGS and SFGS, the MPS includes a 2-point impression score during quiet time. It has been noted [39] that the loss of facial function and the resulting deformity caused by facial palsy often lead to social impairment and mental health issues, such as anxiety, depression, and social stigma. Furthermore, the MPS assessment process is simpler compared to the SFGS and features a scoring range that falls between the HBGS and SFGS, with a total score of 20. The findings of this study suggest that the MPS is comparable to the HBGS and SFGS in terms of efficacy and reactivity, demonstrating a high correlation and sensitivity with the HBGS. Therefore, considering all factors, MPS may be a more eclectic assessment method.

### 4.3. Help to clinical practice

First, we found that HBGS, SFGS, and MPS are all effective for assessing facial nerve function in patients with Bell’s palsy. Their performance in terms of reactivity and efficacy is nearly equivalent. However, considering their specific scoring criteria and the complexity of the assessment process, we believe that the HBGS may be more suitable for rapid screening in primary care settings. The SFGS is better suited for clinical research or cases that require more detailed management, such as after facial nerve transplantation [40,41]. The MPS is appropriate for routine outpatient assessments.

Secondly, the optimal cut-off value can effectively facilitate the application of the scale in clinical practice, allowing for the straightforward division of patients into high- and low-risk groups. In this study, we established HBGS ≥ 3, as summarized in previous research [33], as the threshold for severe symptoms. We predicted that the best cut-off values for SFGS and MPS were ≤ 68 points and ≤ 16 points, respectively. In other words, when assessing facial nerve function in patients with Bell’s palsy, SFGS scores ≤ 68 or MPS scores ≤ 16 are indicative of severe conditions or incomplete recovery. However, it is important to note that we did not utilize more objective radiographic measures as criteria; therefore, the conclusions should be interpreted with caution. Future clinical studies that combine objective indicators, such as electromyography, with subjective assessment scales should be conducted to further validate this conclusion.

Finally, with the advancement of artificial intelligence (AI), the field of facial nerve function assessment is poised to embrace new opportunities. Studies have demonstrated [42,43] that AI models developed using clinical grading scales can automate facial nerve grading and subregion analysis by training on clinical marker data, significantly reducing the workload for physicians. With the optimization of algorithms, AI also enhances the efficiency of extracting facial feature points from images or videos [44]. Currently, AI remains in its early stages, and ethical and safety considerations must be thoroughly addressed when applied to facial palsy assessment. Nevertheless, it is anticipated that AI will undoubtedly pave the way for a brighter future in facial nerve function assessment.

### 4.4. Limitations

The study has several limitations. First, we did not compare the differences between the dimensions of the scale, which affects the comprehensiveness of our conclusions. Second, we did not utilize objective electrophysiological indicators, such as electroneurograms or surface electromyography, which may have rendered the results somewhat subjective. Third, Although the scale ratings were averaged from the results of two independent raters, there was still a degree of bias; it is advisable in future studies to have different scales rated by different professionals to minimise the risk of bias. Fourthly, this study did not assess inter- and intra-rater reliability, which may compromise the accuracy, reproducibility, and external validity of the results. Future studies should incorporate such assessments to ensure data consistency and reproducibility. Finally, the sample size was not calculated but was only estimated to be four to five times larger than that of similar studies [42,45], which may weaken the strength of the evidence supporting our findings.

## 5. Conclusion

The HBGS, SFGS, and MPS are all effective tools for assessing facial nerve function in patients with Bell’s palsy. The SFGS, which has a cut-off score of 68, and the MPS, with a score of 16, can be utilized clinically to classify the severity of Bell’s palsy. However, since this study did not employ objective electrophysiological indicators as a measure, the results should be interpreted with caution.

## Supporting information

S1 Fig Original English version of the scale. (PDF)

S2 Fig The Chinese version of the scale was translated from the original English scale. (PDF)

S1 Data Minimal data set. (PDF)
