# Supplementary material for: Evaluation of nerve function after Bell’s palsy based on different facial nerve assessment scales HBGS/SFGS/MPS: A comparative study
Source: PLoS One. 2025 Jun 25;20(6):e0326789. doi: 10.1371/journal.pone.0326789 (PMC12193829; doi:10.1371/journal.pone.0326789)
Supplement: S1 Fig — (PDF) [file pone.0326789.s001.pdf]

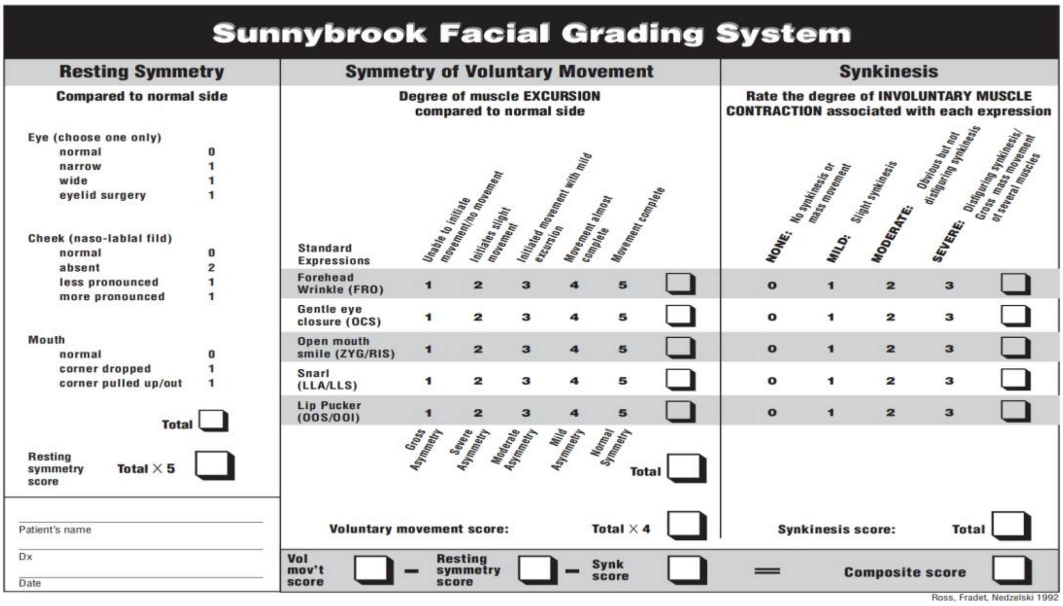

Sunnybrook Facial Grading System

Resting Symmetry

Compared to normal side

**Eye (choose one only)**	
normal	0
narrow	1
wide	1
eyelid surgery	1
**Cheek (naso-labial fild)**	
normal	0
absent	2
less pronounced	1
more pronounced	1
**Mouth**	
normal	0
corner dropped	1
corner pulled up/out	1
**Total**	☐

Resting symmetry score — Total × 5 ☐

Patient's name

Dx

Date

Symmetry of Voluntary Movement

Degree of muscle EXCURSION compared to normal side

Standard Expressions	Unable to initiate movement/no movement	Initiates slight movement	Initiated movement with mild excursion	Movement almost complete	Movement complete	
Forehead Wrinkle (FRO)	1	2	3	4	5	☐
Gentle eye closure (OCS)	1	2	3	4	5	☐
Open mouth smile (ZYG/RIS)	1	2	3	4	5	☐
Snarl (LLA/LLS)	1	2	3	4	5	☐
Lip Pucker (OOS/OOI)	1	2	3	4	5	☐
	Gross Asymmetry	Severe Asymmetry	Moderate Asymmetry	Mild Asymmetry	Normal Symmetry	Total ☐

Voluntary movement score: Total × 4 ☐

Synkinesis

Rate the degree of INVOLUNTARY MUSCLE CONTRACTION associated with each expression

NONE: No synkinesis or mass movement	MILD: Slight synkinesis	MODERATE: Obvious but not disfiguring synkinesis	SEVERE: Disfiguring synkinesis/ Gross mass movement of several muscles	
0	1	2	3	☐
0	1	2	3	☐
0	1	2	3	☐
0	1	2	3	☐
0	1	2	3	☐

Synkinesis score: Total ☐

Vol mov't score ☐ − Resting symmetry score ☐ − Synk score ☐ = Composite score ☐

Ross, Fradet, Nedzelski 1992

Figure S1. Original English version of the scale.
