# Supplementary material for: Evaluation of nerve function after Bell’s palsy based on different facial nerve assessment scales HBGS/SFGS/MPS: A comparative study
Source: PLoS One. 2025 Jun 25;20(6):e0326789. doi: 10.1371/journal.pone.0326789 (PMC12193829; doi:10.1371/journal.pone.0326789)
Supplement: S2 Fig — (PDF) [file pone.0326789.s002.pdf]

Sunnybrook 多伦多面神经评定系统(SFGS)

静态时与健侧比较（每项评分只能选择一种）			基线评估评定日期:	终点评估评定日期:
眼（睑裂）	正常	0		
	缩窄	1		
	增宽	1		
	做过眼睑整形手术	1		
颊（鼻唇沟）	正常	0		
	消失	2		
	不明显	1		
	过于明显	1		
嘴	正常	0		
	口角下垂	1		
	口角上提	1		
静态分=总分×5				

与健侧相比随意运动的对称性							
标准表情	无运动(完全不对称)	轻度运动	有运动但有错乱的表情	运动接近对称	运动完全对称	基线评分	终点评分
抬额头	1	2	3	4	5		
轻轻闭眼	1	2	3	4	5		
张嘴微笑	1	2	3	4	5		
耸鼻	1	2	3	4	5		
唇吸吮	1	2	3	4	5		
随意运动分=总分×4							

联动分级						
标准表情	没有联动	轻度联动	明显联动但无毁容	严重的毁容性联动	基线评分	终点评分
抬额头	0	1	2	3		
轻轻闭眼	0	1	2	3		
张嘴微笑	0	1	2	3		
耸鼻	0	1	2	3		
唇吸吮	0	1	2	3		
联动分=总分						
最 后 得 分						
评价人签名						

注释：最后得分=随意运动分-静态分-联动分；Sunnybrook(多伦多）面神经评定系统得分在0-100分，分值越高，表示面神经功能越好。

Figure S2. The Chinese version of the scale was translated from the original English scale.
