# Supplementary material for: Evaluation of nerve function after Bell’s palsy based on different facial nerve assessment scales HBGS/SFGS/MPS: A comparative study
Source: PLoS One. 2025 Jun 25;20(6):e0326789. doi: 10.1371/journal.pone.0326789 (PMC12193829; doi:10.1371/journal.pone.0326789)
Supplement: S1 Data — (PDF) [file pone.0326789.s003.pdf]

Number	HB-Before	HB-after	SFGS-Before	SFGS-after	MPS-Before	MPS-after
1	5	3	16	57	4	12
2	5	3	8	50	3	13
3	5	3	13	47	3	9
4	5	3	18	50	5	13
5	5	4	11	36	2	7
6	5	4	10	28	2	6
7	5	3	14	49	2	9
8	5	3	24	54	4	10
9	3	2	50	84	9	17
10	5	4	9	27	2	5
11	5	2	18	72	5	16
12	4	4	27	27	7	7
13	4	3	54	80	9	17
14	4	3	46	80	7	16
15	4	3	51	88	9	16
16	4	3	59	96	8	17
17	4	2	42	84	5	17
18	5	4	14	32	3	7
19	4	2	41	88	7	16
20	5	2	33	96	4	18
21	3	2	63	84	11	17
22	4	2	45	80	7	17
23	3	2	63	88	12	16
24	4	3	22	50	5	9
25	4	2	18	55	5	11
26	5	2	34	96	6	18
27	5	3	32	67	6	14
28	5	4	18	36	4	8
29	5	3	16	51	4	10
30	4	2	54	88	12	15
31	4	1	55	100	11	20
32	5	3	42	55	7	13
33	5	3	28	51	4	11
34	4	3	27	67	7	14
35	5	3	20	67	5	13
36	4	2	43	96	6	19
37	4	2	21	59	6	14
38	5	4	18	40	4	8
39	5	3	28	67	5	9
40	5	2	14	84	4	17
41	5	2	20	68	5	14
42	3	1	66	100	13	20

43	5	1	19	100	3	20
44	5	1	32	100	5	20
45	5	1	24	100	6	20
46	5	1	22	100	4	20
47	5	2	13	88	3	19
48	5	1	19	100	4	18
49	5	3	10	58	5	11
50	3	1	59	100	11	20
51	5	4	13	21	5	8
52	5	2	12	88	4	17
53	5	3	9	51	4	11
54	5	2	21	88	5	18
55	5	3	18	58	4	9
56	5	2	10	88	3	15
57	5	3	21	77	6	17
58	5	2	21	84	5	16
59	4	2	38	80	8	16
60	5	3	13	51	3	11
61	5	3	15	51	4	10
62	5	3	20	45	5	10
63	4	2	30	84	8	14
64	5	3	20	49	5	11
65	5	3	28	77	3	14
66	5	3	16	58	5	15
67	5	2	15	92	5	17
68	5	3	20	67	6	15
69	4	2	49	92	7	16
70	5	3	29	55	4	11
71	5	3	22	61	5	13
72	5	2	25	88	3	18
73	5	3	20	52	4	12
74	5	3	21	54	5	12
75	5	3	16	67	4	14
76	5	3	24	53	5	11
77	3	1	46	100	10	20
78	5	2	23	85	4	16
79	5	3	26	57	4	11
80	5	3	20	50	5	12
81	4	1	48	100	9	20
82	4	2	47	88	9	17
83	5	2	16	76	5	13
84	4	3	41	67	7	13
85	5	3	11	53	3	15

86	4	2	43	88	7	17
87	5	2	23	92	4	17
88	5	3	23	59	6	11
89	5	4	15	32	4	11
90	5	3	15	61	6	12
91	5	3	27	51	4	14
92	5	3	16	84	5	17
93	5	1	15	100	5	20
94	4	2	43	76	7	16
95	5	2	29	76	5	16
96	5	2	23	88	5	18
97	5	4	20	49	4	7
